# Bee Bread: A Promising Source of Bioactive Compounds with Antioxidant Properties—First Report on Some Antimicrobial Features

**DOI:** 10.3390/antiox13030353

**Published:** 2024-03-15

**Authors:** Cornelia-Ioana Ilie, Angela Spoiala, Elisabeta-Irina Geana, Cristina Chircov, Anton Ficai, Lia-Mara Ditu, Eliza Oprea

**Affiliations:** 1 Department of Science and Engineering of Oxide Materials and Nanomaterials, National Centre for Micro and Nanomaterials and National Centre for Food Safety, Faculty of Chemical Engineering and Biotechnologies, National University of Science and Technology Politehnica Bucharest, 060042 Bucharest, Romaniacristina.chircov@upb.ro (C.C.); 2 National R&D Institute for Cryogenics and Isotopic Technologies (ICIT), 240050 Râmnicu Vâlcea, Romania; irina.geana@icsi.ro; 3 Academy of Romanian Scientists, 010719 Bucharest, Romania; 4 Department of Botany and Microbiology, Faculty of Biology, Research Institute, University of Bucharest, 060101 Bucharest, Romania; lia-mara.ditu@bio.unibuc.ro (L.-M.D.);

**Keywords:** bee bread, phenolic compounds, antioxidants, antimicrobial activity, antibiofilm activity

## Abstract

Bee bread has received attention due to its high nutritional value, especially its phenolic composition, which enhances life quality. The present study aimed to evaluate the chemical and antimicrobial properties of bee bread (BB) samples from Romania. Initially, the bee bread alcoholic extracts (BBEs) were obtained from BB collected and prepared by *Apis mellifera carpatica* bees. The chemical composition of the BBE was characterized by Fourier Transform Infrared Spectroscopy (FTIR) and the total phenols and flavonoid contents were determined. Also, a UHPLC-DAD-ESI/MS analysis of phenolic compounds (PCs) and antioxidant activity were evaluated. Furthermore, the antimicrobial activity of BBEs was evaluated by qualitative and quantitative assessments. The BBs studied in this paper are provided from 31 families of plant species, with the total phenols content and total flavonoid content varying between 7.10 and 18.30 mg gallic acid equivalents/g BB and between 0.45 and 1.86 mg quercetin equivalents/g BB, respectively. Chromatographic analysis revealed these samples had a significant content of phenolic compounds, with flavonoids in much higher quantities than phenolic acids. All the BBEs presented antimicrobial activity against all clinical and standard pathogenic strains tested. *Salmonella typhi*, *Candida glabrata*, *Candida albicans*, and *Candida kefyr* strains were the most sensitive, while BBEs’ antifungal activity on *C. krusei* and *C. kefyr* was not investigated in any prior research. In addition, this study reports the BBEs’ inhibitory activity on microbial (bacterial and fungi) adhesion capacity to the inert substratum for the first time.

## 1. Introduction

One of the oldest traces of bee products comes from Spain, Cave Spider, where a rock painting was discovered in 1919. The painting dates from the Neolithic Age and illustrates a human taking honey from wild bees [1]. There are historical beliefs that state the Greeks considered honey and bee products the foods of kings, providing youth and life [2]. Other evidence of the history of beekeeping dates from the 10th century, when the Arab traveler Ibrahim ibn Yaqub wrote about Poland as being a country rich “in food, meat, honey, and arable lands” [3].

From all the statements and oldest records from ancient times, beliefs at present consider that a healthy lifestyle will lead to better health [4]. Therefore, it is believed that ancient people used bee products to treat diverse diseases, which included wounds, ulcers, and bowel problems [5]. The bee products, especially (bee pollen–BP, BB, propolis, honey, bee venom, etc.), were found to have strong effects on physical and mental health. Nevertheless, they have protective and therapeutic effects against several diseases. Moreover, researchers have taken a particular interest in BP and BB as a result of the strong relationship between their nutritional values and their therapeutic properties [6,7,8,9,10,11].

Bee bread is an apitherapeutic product resulting from the lactic fermentation of pollen harvested by honeybees. Collected BP is mixed with digestive enzymes from bees’ salivary glands and stored in a honeycomb with a wax and honey layer. BP and BB have similar metabolic and nutritional values, containing macro- and micronutrients, phenolic acids, and polyphenols [4]. The nectar and pollen from plant flowers provide the BB nutritional value. Recent studies have presented a relevant/direct connection between their composition, physicochemical properties, and therapeutic role in human health [12,13,14,15].

In their work, Bakour et al. [15] studied BB’s chemical composition and bioactive properties and clinched, as expected, that the chemical composition dictates antioxidant properties. Many researchers have shown that BB is a natural source of antioxidants such as PCs, coenzyme Q_10_, etc. [16]. Additionally, BB is an excellent natural source of bioactive compounds due to its high nutritional value, which is variable according to its botanical origin and geographical region [17,18]. Kieliszek et al. [4] showed that the dietary value of BB is much higher than BP, and it is more digestible due to the higher amount of amino acids and easily assimilated sugars. Similar studies have reported the BB content in water and the presence of significant amounts of proteins, amino acids, carbohydrates, lipids, vitamins, minerals, and polyphenols [16,19,20,21].

The most common PCs in bee products are flavonoids and phenolic acids [22,23]. Studies have discovered the significant role of PCs reflected in the BB’s therapeutic and biological properties [13,24]. As discovered, flavonoids found in BB have important medico-pharmacological applicability in antioxidant, anticancer, anti-inflammatory, neuroprotective, hormone regulation, and antidiabetic activities [4,24,25].

Likewise, BB consists of rich quantities of carotenoids, a group of antioxidant compounds responsible for its colors (red, yellow, orange, brown, etc.) and depends on its botanical origin [6]. Carotenoids sustain cellular growth and regulation and can prevent diseases such as cancer [26]. Depending on the botanical source, BB contains several fat and water-soluble vitamins (A, D, E, K, C, and B complex). Vitamin E is an important antioxidant vitamin found in BB. Additionally, it has diverse biological properties, such as antitumoral, immunostimulatory, anti-inflammatory, and neuroprotective [27].

The BB (as BP) presents anti-inflammatory, antifungal, anticancer, antimicrobial, antioxidant, and gastroprotective properties, as well as neuroprotective and anti-aging activities [24,25,28,29,30,31]. In recent years, there has been a persistent interest regarding the connection between the antimicrobial properties of bee products and antimicrobial resistance to pathogens. Therefore, researchers and scientists have demonstrated that BB possesses microorganisms and bioactive compounds that can provide them with the properties of a probiotic and prebiotic product [32,33,34]. BB is produced through the lactic bacteria fermentation process using microorganisms such as *Lactobacillus* spp. and *Saccharomyces* spp. [35]. In a similar context, Toutiaee et al. [36] reported the isolation of *Bacillus* sp. with probiotic properties, whose bacteria content made BB a considerable source of probiotics. According to Bleha et al. [37], the study’s results obtained from the microbial growth assay show that BB can be used for symbiotic construction.

Other findings reported BB as a powerful biomarker/biomonitor, according to Schaad et al. [38], who quantified pesticides in BB samples collected from honeybees in an agricultural environment in Switzerland. The study results provided a significant basis for monitoring pesticide contamination [39].

From our knowledge, most articles focused on a single BB sample’s physico-chemical and biological characterizations. Only two published studies analyzed BB samples collected from different geographical areas of Greece [28] (18 samples) and Romania [17] (13 samples from other regions compared to those selected in the present study). If in the first study, TF, TP, antioxidant (DPPH and ABTS), and antimicrobial analyses of BB were carried out, in the second, the nutritional properties (fatty acids, proteins, amino acids, and free saccharides) and phenolic compounds were highlighted (flavonol glycoside derivatives, by HPLC), without addressing the antimicrobial analysis. In addition, while both previously mentioned studies analyzed BB samples from local producers, in the case of the studies in this paper, both BB samples from local producers and commercially purchased samples were used. Another comparative study was carried out on 15 samples of Colombian BB [40], and it only aimed to establish protein and lipid levels. Still, other research has been carried out on BB collected from various geographical areas but on a smaller number of samples, such as Ukraine (five samples) [41], Lithuania (nine samples) [42], Portugal (six samples) [43], or Turkey (five samples) [44].

The present study illustrates the palynological analysis, chemical composition, and antioxidant and antimicrobial activities of twelve BB samples. First, botanical origin analysis using scanning electron microscopy (SEM) and a light microscope (LM) was performed. Chemical composition was determined using FTIR spectroscopy, and the PCs were evaluated using spectrophotometric (total phenolic compounds and total flavonoids) and chromatographic methods. Additionally, antioxidant capacity was determined using a spectrophotometric assay. The antimicrobial activity of the BBEs was qualitative and quantitatively evaluated on some pathogenic strains (standard and clinical). In addition, the novelty of this study consists of the inhibitory activity of microbial (bacterial and fungi) adhesion capacity to the inert substratum induced by BBEs, as well as their antifungal activity on *Candida krusei* and *Candida kefyr*, which are for the first time reported.

## 2. Materials and Methods

### 2.1. Materials

**Figure 1:**
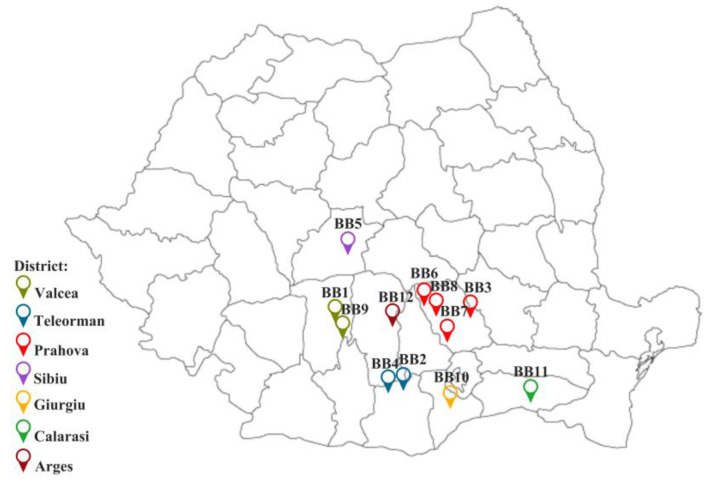
Distribution of geographical origin of BB samples. Created with ConceptDraw Diagram.

The BB samples were provided by Romanian beekeepers between the spring and summer of 2022 and were deposited at −45 °C. Some samples were extracted from the honeycombs by us and immediately stored in a freezer (**BB1**-**BB3**, **BB12**); others were already removed by the beekeepers with special devices (**BB4**-**BB11**). The geographical origin of BB samples is presented in Figure 1. The apiaries were distributed in seven districts of Romania: Arges, Calarasi, Giurgiu, Prahova, Sibiu, Valcea, and Teleorman.

The BBs come from pollen harvested by *Apis mellifera carpatica* bees from wild flora and house plants, and for these reasons, a palynological analysis was carried out to establish its botanical origin accurately.

### 2.2. Reagents

Ethanol, glycerol, Folin–Ciocâlteu phenol reagent, sodium carbonate, aluminum chloride, 2,2′-azino-bis (3–ethylbenzothiazoline-6-sulfonate), potassium persulfate, phenolic standards, Nutrient Broth No. 2 (NB), Sabouraud Glucose Agar (Sab) with chloramphenicol, acetic acid (AcA), and crystal violet (CV) were acquired from Sigma-Aldrich (Darmstadt, Germany). Methanol, formic acid, and gallic acid were purchased from Merck (Darmstadt, Germany). All reagents presented high analytical purity, and the strains were part of the Microorganisms Collection of the Department of Microbiology, Faculty of Biology and Research Institute of the University of Bucharest.

### 2.3. Palynological Analysis

The palynological identification was performed using scanning electron microscopy (SEM) and a light microscope (LM). A total of 2 g of each BB, corresponding to ~500 pollen grains, was considered representative of botanical identification [45]. A quantity of 15 mg of BB was hydrated in 1.5 mL of deionized water. Each suspension was vortexed for 1 min at 20 rpm and immediately spread in 3 equal parts on microscope slides/smooth adhesive surfaces on an aluminum stub.

The SEM images for the determination of the size and morphology of the pollen grains were recorded using a Quanta Inspect F50 (FEI Company, Eindhoven, The Netherlands) scanning electron microscope equipped with a field emission gun electron (FEG) with a 1.2 nm resolution and an energy-dispersive X-ray spectrometer with an MnK resolution of 133 eV. Before the analysis, the BB samples were coated with gold.

The Primostar 3 KMAT Microscope was used for LM analysis. The images were recorded using an Axiocam 208 colour Camera (Carl Zeiss Meditec, Jena, Germany) and Zen Blue 3.4 software. The slides with BB suspensions were dried at room temperature. Glycerol was liquefied at 40 °C, and a drop (100 μL) was put on the slide, which was covered with a thin slide [28].

The pollen grains were identified using online palynological databases (paldat.org and pollenatlas.net (accessed on 1 February 2023)) [46,47] and recent studies [48,49,50,51].

### 2.4. Bee Bread Extract (BBE) Preparation

The extractions were performed using a method described in a previous study [9]. 1.25 g BB was heated for 10 min at 40 °C with ethanol 70% (*v*/*v*) using an ultrasonic bath (Germany, Elmasonic). This process included more steps, such as vortexing (2600 rpm, 3 min), ultrasonication, and centrifugation (4000 rpm at 4 °C, 10 min). The BBEs’ final volume was 25 mL for each sample, and the extracts were stored in closed bottles at −45 °C till analysis.

### 2.5. Chemical Characterization of BBE

#### 2.5.1. Fourier Transform Infrared Spectroscopy (FTIR)

The FTIR measurements were performed using a Nicolet iS50R spectrometer (Thermo Fisher Scientific, Waltham, MA, USA). The spectra were recorded at room temperature using the attenuated total reflection (ATR) (Thermo Fisher Scientific, Waltham, MA, USA), with 32 scans between 4000 and 400 cm^−1^ at a resolution of 4 cm^−1^, with the scanning time being 47 s [52].

#### 2.5.2. Determination of Total Phenols Content (TPC)

The TPC was performed using the Folin–Ciocâlteu method [53]. A total of 0.5 mL of BBE or standard (gallic acid), 5 mL of Folin–Ciocâlteu reagent (diluted 10 times in distilled water), and 4 mL of 1 M of sodium carbonate were homogenized, and the absorbance was measured after 15 min (min) at 746 nm using a Shimadzu UV-1800 spectrophotometer (Kyoto, Japan). A calibration curve was plotted with standard solutions of gallic acid with concentrations varying between 5 and 150 mg/L. TPC was expressed as milligrams (mg) of gallic acid equivalents (GAEs)/gram (g) of the sample [9,53,54,55].

#### 2.5.3. Determination of Total Flavonoids Content (TFC)

The BBEs’ TFC was determined by the aluminum chloride method described in a previous study [9]. The absorbance was measured at 430 nm (spectrophotometer presented in Section 2.5.2), and the TFC was expressed in mg quercetin (QE)/g BB; it was calculated by applying the calibration curve obtained for concentrations of quercetin varying between 0 and 0.12 mg/mL [9,54].

#### 2.5.4. Phenolic Compound Analysis by UHPLC-MS/MS

The measurements were performed using a Q Exactive™ Focus Hybrid Quadrupol OrbiTrap mass spectrometer (ThermoFisher Scientific, Darmstadt, Germany) equipped with heated electrospray ionization (HESI) (ThermoFisher Scientific), coupled with an UltiMate 3000 UHPLC system consisting of a quaternary pump, column oven, and autosampler (ThermoFisher Scientific). Chromatographic separation of phenolic compounds was performed on two columns: Accuacore PFP (50 mm × 2.1 mm, 2.6 μm) and Accuacore PFP (100 mm × 2.1 mm, 2.6 μm) from Thermo Fisher Scientific under the gradient elution of solvent A (water with 0.1% formic acid) and solvent B (methanol with 0.1% formic acid). Chromatographic and mass spectrometric parameters were set according to Ciucure and Geană [56].

Phenolic acids and flavonoids from BBEs were identified according to mass spectra, accurate mass, and characteristic retention time against external standard solutions analyzed under the same conditions. Data-dependent scans with collision-induced dissociations (CIDs) set between 15 and 60 eV were performed for fragmentation studies to confirm each analyzed phenolic compound. Xcalibur software (Version 4.1) was used for instrument control, data acquisition, and data analysis. The ChemSpider internet database of accurate MS data (www.chemspider.com, accessed on 17 November 2023) was used as a reference library to identify compounds of interest [9]. For quantification, a calibration was performed via serial dilution with methanol of the standard mixture of a concentration of 100 mg/L, covering a calibration range between 0 and 10 mg/L [56]. The results are expressed as µg/g of the BB sample.

#### 2.5.5. Determination of Trolox Equivalent Antioxidant Capacity (TEAC)

This method was performed by highlighting the neutralization potential of the cation radical ABTS^•+^ (2,2′-azino-bis (3–ethylbenzothiazoline-6-sulfonate), expressed as Trolox equivalents. The TEAC method is based on the capacity of the BBE to discolor the blue–green chromophore radical ABTS^•+^. All steps were described in previous studies [9,57,58], and the spectrophotometric readers were performed at 734 nm. The results are expressed in millimoles of Trolox equivalent (mmol Trolox)/g BB.

### 2.6. Methods Applied in the Biological Activity of BBE

#### 2.6.1. Qualitative Assessment of the Antimicrobial Activity

The antimicrobial assays were assessed with standard strains (*Enterococcus faecalis* ATCC 29212, *Staphylococcus aureus* ATCC 25923, *Escherichia coli* ATCC 25922, and *Pseudomonas aeruginosa* ATCC 27853) and clinically isolated strains (*Bacillus subtilis*, *Salmonella typhi* 14023, *Candida albicans* 1688, *Candida glabrata, Candida kefyr*, and *Candida krusei*).

The antimicrobial properties of BBE samples (the same extracts characterized previously) were assessed by a spot diffusion assay [9,59,60] and according to the Clinical Laboratory Standards Institute [61]. Bacterial and yeast suspensions (1.5 × 10^8^ CFU/mL and 3 × 10^8^ CFU/mL, respectively) were obtained from 24 h cultures on NB and Sab media. Petri plates with the specific media were seeded with inoculums, and each 20 µL of each BBE was spotted. BBE samples contained ethanol, so an ethanol-based control (C_Et_) was used. The negative control was considered the sterile medium, and the positive control was the NB/Sab medium inoculated with microbial suspensions. After diffusion, the dishes were incubated at 37 °C for 24 hours (h) for bacteria and 48 h for yeast strains.

#### 2.6.2. Quantitative Assessment of the Antimicrobial Activity

The minimum inhibitory concentration (MIC) assessment was performed using an adapted binary serial microdilution standard assay [9,60,61] in liquid media utilizing 96-well microtiter plates. From every BBE sample, serial two-fold microdilutions were realized in 150 μL of corresponding broth medium seeded with the standard inoculum. Also, a control was performed with ethanol (C_Et_). The negative and positive controls were performed following the identical steps already detailed. The plates were incubated at 37 °C for 24 h. The MIC values were determined by visual and spectrophotometric analyses measuring the absorbance at 620 nm via the BIOTEK SYNERGY-HTX ELISA multi-mode reader (VT, USA).

#### 2.6.3. Semiquantitative Assessment of the Microbial Adherence to the Inert Substratum

The biofilm development on the inert substratum was assessed utilizing the identical serial two-fold microdilution method [9]. Following 24 h of incubation, the media from dishes (containing binary dilutions of the BBEs) was removed, the wells were washed three times with sterile physiological buffer saline (PBS), and the bacterial cells adhered to the walls were fixed with methanol (5 min) and tinted with 1% CV (15 min). The stained biofilm was resuspended with 33% AcA, and the absorbance was measured at 490 nm [60,61].

### 2.7. Statistical Analysis

The data results were statistically analyzed with GraphPad Prism 10.2 from GraphPad Software, San Diego, CA (USA). The results are expressed as ±SD (standard deviation) and analyzed using a one-way analysis of variance (one-way ANOVA) and Tukey’s multiple comparisons test according to the method of the experiments performed. The differences between groups were considered statistically significant when the *p*-value was <0.05.

Principal component analysis (PCA) and hierarchical cluster and heat map analysis (HMA) were performed using Microsoft Excel 2010, Microsoft, Redmond, Washington, DC (USA) and XLSTAT Add-in (15.5.03.3707 software version) by Addinsoft, New York, NY (USA). The chord diagram established by the correlation between botanical families and BB samples was analyzed with OriginPro version 2024 from OriginLab Corporation, Northampton, MA (USA).

## 3. Results and Discussions

### 3.1. Palynological Analysis

**Figure 2:**
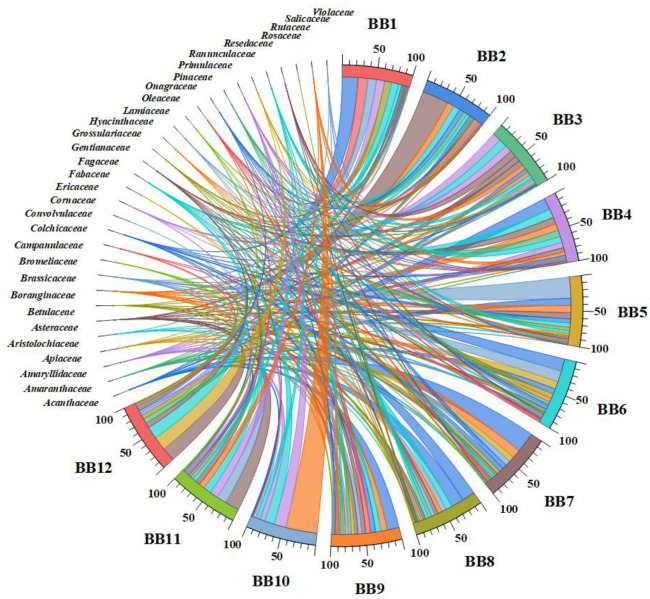
Chord diagram based on the relationship between botanical families and BB samples.

**Figure 3:**
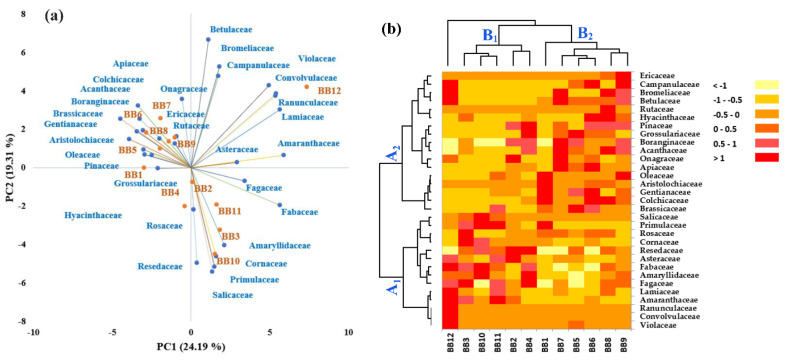
Discrimination of bee bread samples based on a palynological analysis. (**a**) Principal component analysis and (**b**) hierarchical cluster and heat map analysis.

BBs’ taxonomic/botanical assignments were performed using LM and SEM, and Figure 2 and Figure 3 represent the comprehensive results. As a result of the microscopic study, the LM is considered mandatory for determining the relative abundance of species identified. Likewise, the high-resolution SEM images are helpful for the size and morphology determination of the pollen grains, especially the surface pattern, polarity, shape, or dispersal units [62].

The data results of the botanical identification presented in Figure 2 indicate 31 families identified and a detailed interconnection between the plant families and BB samples. Also, a high number of plant families and the relative abundance percentages (%) for each BB sample can be observed. For example, **BB8** presented pollen grains from an extensive list of plant families (24), and **BB6** and **BB7** were from 22. Likewise, in **BB1**, **BB3**, **BB5**, and **BB9** samples, 21 plant families were identified. **BB10** was the least varied sample, containing pollen grains from 9 plant families, with the *Salicaceae*, *Primulaceae*, and *Fabaceae* as the dominant families.

BP from the *Fabaceae* family is present in all BB samples, which range from 3.00 to 18%, and *Acanthaceae*, *Amaryllidaceae*, and *Fagaceae* plant families are present in 11 BB samples. Likewise, the families predominantly in the 10 BB samples are *Amaranthaceae*, *Asteraceae*, *Boranginaceae*, *Onagraceae*, and *Primulaceae* (Figure 2). Otherwise, the high relative abundance is for pollen from the *Salicaceae* family, with 47% in **BB10**. BP from the *Asteraceae* family is predominant in **BB2** (39%), followed by the *Brassicaceae* family in **BB5** and the *Acanthaceae* family in **BB7** (30%). The lowest percentages (<1.5%) are presented in BP which comes from the *Convolvulaceae* (**BB12**) and *Rutaceae* (**BB8**) families.

Multivariate statistical analysis was performed on the plant families identified in BBs to differentiate and group/cluster the BB samples based on the palynological analysis. The PCA plot of the plants’ taxonomic assignments from BP (Figure 3a) showed a clear discrimination of **BB12** and some provenience families (see the upper right part of the graph), which are correlated with the relative abundance presented in Figure 2. Likewise, the high frequencies of *Amaryllidaceae*, *Cornaceae*, *Fabaceae*, *Fagaceae*, *Primulaceae*, and *Salicaceae* in pollen from **BB2, BB3**, **BB10,** and **BB11** are confirmed in the PCA plot. Particularly, **BB1** and **BB4** are grouped on the left side of Figure 3a, and *Resedaceae*, *Rosaceae*, *Hyanthaceae*, and *Grossulariaceae* are the representative/specific families from which the pollen of these samples originates. The other families are associated with **BB5**, **BB6**, **BB7**, **BB8,** and **BB9**.

In order to confirm the PCA assay and Chord diagram and differentiate the BB samples as much as possible, hierarchical clustering and heat map assays were performed. According to Figure 3b, the data obtained confirm the previous results. As in Figure 3a, the BB samples were grouped into two main groups (B_1_ and B_2_), and this highlighted a discrimination of **BB12**. The heat map combined with the clusters also expressed a snapshot of the botanical origin of BB samples and was represented by a main group/cluster, which was fused in two subclusters (A_1_ and A_2_). Group A_1_ corresponds to the right side of the PC1-PC2 graph, and A_2_ to the left side.

Previous studies identified similar botanical origins of pollen grains in BB or BP from Romania [9,17,63,64,65,66]. This study analyzed BB samples from different areas of Romania, with quite varied vegetation, and, for this reason, vast plant botanical families were identified. Furthermore, significant differences in dominant plant families identified depending on the geographical origin of the BB samples were not observed.

### 3.2. Chemical Composition of BBE

The complex composition of BB can vary widely due to many factors, such as plant and bee species, geographical area, seasonal changes, fermentation strains, beekeeper activities, etc. [67,68]. The alcoholic extracts (BBEs) were analyzed to establish the samples’ chemical compositions.

#### 3.2.1. FTIR Spectroscopy

The FTIR spectra for all BB samples were measured between 4000 and 400 cm^−1^, as illustrated below.

**Figure 4:**
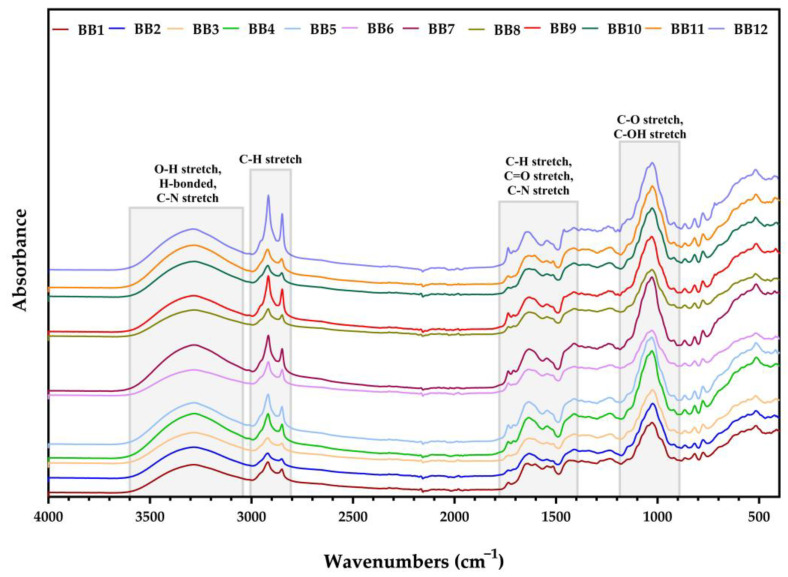
FTIR spectra for BB.

Figure 4 shows that all BB samples presented similar FTIR spectra, which were characterized in previous studies [65,66,69]. Also, all BB samples shared comparable adsorption bands with minor spectral differences.

In the region of 3600–3050 cm^−1^, the stretching vibrations of O-H and H bonds, which correspond to the presence of water, can be seen [70]. Likewise, in the same intervals, a functional group of amines I and II and amides also exist, which suggests the presence of amino acids and proteins (previously identified in BB) [66]. Between the interval of 3000–2800 cm^−1^, the peaks were assigned to the symmetric and asymmetric stretching of the C-H groups in carbohydrates and lipids contained in all BB samples [71,72]. Likewise, other peaks related to lipids were observed between the interval of 1790–1400 cm^−1^, which were attributed to C=O stretching in the ester bond and C-H deformation vibrations of lipids (triglycerides, phospholipids, etc.) [72,73]. Moreover, the C=O and C=C stretching vibrations occurred due to the presence of PCs (sesquiterpenes, phenolic acids, flavonoids, stilbenoids, etc.) [70]. In the same interval, C-N stretching vibrations from amide II were observed [72]. The last gap, 1390–900 cm^−1^, showed a large peak corresponding to C-O vibrations from carbohydrates and fatty acids, and C-OH groups from polyphenols [66,70,73].

#### 3.2.2. Determinations of TPC, TFC, and TEAC

TPC, TFC, and TEAC results are presented in Table 1 as mean value ± SD.

The results presented in Table 1 show that **BBE8** and **BBE10** presented the highest values of TPC (18.30 ± 0.029 and 18.30 ± 0.051 mg GAE/g BB). Correlating the TPC and TFC results with TEAC values is also sometimes difficult. Still, even if the phenolic acids and flavonoids are the primary compounds to determine antioxidant activity, other biomolecules can influence this (stilbenoids, sesquiterpenes, carotenoids, proteins, etc.) [74,75,76,77,78,79]. The antioxidant activity of BBE (corresponding to the TEAC method) had values ranging from 0.02 ± 0.005 to 0.07 ± 0.020 mmol Trolox/g BB.

The TPC, TFC, and antioxidant activity depend upon the plant species from which they are derived. Moreover, Mărgăoan et al. [64] reported the highest antioxidant activity of multifloral BP, with the TEAC method, was for samples that were predominantly from plants of *Asteraceae*, *Brassicaceae*, *Fabaceae*, and *Fagaceae* plant families. Likewise, the highest levels of TPC were attributed to *Rosaceae* and *Brassicaceae*. In contrast, the TFC was highest in BB samples containing BP from *Lamiaceae*.

Gercek et al. [80] obtained a TFC of 79.21 mg QE/g BP from samples originated from the *Asteraceae*, *Fabaceae*, *Campanulaceae*, *Cistaceae*, and *Rosaceae* plant families. In our study, the TFC for **BBE11** was 0.95 ± 0.011 mg QE/g BB and **BBE12** was 0.85 ± 0.500 mg QE/g BB, and according to the palynological assay, contained pollen from *Asteraceae* (~21%), *Fabaceae* (12% and 18%), and *Campanulaceae* family plant species (2% for **BBE12**). The **BBE3** sample presented the highest TFC (1.86 ± 0.516 mg QE/g BB), probably due to the high polyphenols content of plants that bloom in early spring [9] and belong to families like *Acanthaceae*, *Asteraceae*, *Colchicaceae*, *Cornaceae*, *Fabaceae*, *Fagaceae*, *Onagraceae*, *Primulaceae*, *Ranunculaceae*, *Resedaceae*, *Salicaceae*, etc. Furthermore, Jara et al. [81] reported that the *Acanthus mollis* (*Acanthaceae*) flower presented a high content of polyphenols and antioxidant activity. Significant levels of PCs and antioxidant activity were also demonstrated in BP from the *Oleaceae* family [82], which was present the most in **BBE1**.

#### 3.2.3. Phenolic Compound Profiles by UHPLC-DAD-ESI/MS

A total of 24 compounds, including 9 phenolic acids, 13 flavonoids and derivatives, and stilbene *t*-resveratrol were unambiguously identified, and sesquiterpene abscisic acid was quantified in the BBE. The quantitative results of the individual phenolic compounds in BB are presented in Table 2.

According to the quantitative data, flavonoids were quantified in higher amounts compared with phenolic acids, being in concordance with the TPC and TFC contents of bee bread measured by UV–Vis spectrophotometric methods (Table 1) and literature data [71]. Moreover, the extracts contained other PCs, like rutin, hesperidin, resveratrol, and abscisic acid (ABA). Overall, the samples had significant quantities of phenolic acids, like *p*-coumaric acid, gallic acid, caffeic acid, and cinnamic acid. High contents could be observed for quercetin, kaempferol, isorhamnetin, and ABA. 

The BP fermentation in the BB formation process changed the bioactive compounds’ amounts and chemical profiles to increase their bioavailability and bioaccessibility [83,84,85]. Also, lactic fermentation by bacteria and/or yeast degrades the pollen wall and releases PCs, which are associated with carbohydrates or proteins [84,86]. Additionally, Zhang et al. [87] reported that the contents of flavonoids in fermented BP increased up to 144.66-fold/units, and of phenolic acids only up to 28.9-fold compared to unfermented BP. The flavonoid and phenolic acid glycosides significantly decreased (in some cases, disappeared), which can be explained by the heteroside hydrolyzation into their aglycone forms due to the microbial fermentation process [88]. Among flavonoid heterosides, the BBEs tested in this study contained only rutin (quercetin-3-rutinoside) and hesperidin (hesperetin-7-rutinoside), while Bayram et al. [44] identified only rutin. In addition, probiotic fermentation can affect the phenolic acids, which, through metabolization in small compounds (like 4-ethyl catechol, dihydrocaffeic acid, etc.), provide more biological properties for bees and humans [84].

According to Table 2, **BBE12**, **BBE2**, **BBE9,** and **BBE8** had the highest concentrations of phenolic acids and flavonoids (in this order), but considering the resveratrol and ABA, **BBE9** showed the highest content in PCs. Significant ABA contents can be observed in the samples, especially for **BBE9**, **BBE2**, **BBE3**, **BBE7**, and **BBE8**. The phytohormone ABA, with multiple regulatory functions in plants, comes from flower nectar. Likewise, ABA enhances the immune system, cold stress tolerance, growth, and prevalence of the nosemosis (Nosema disease) of *Apis mellifera* bees [68,89,90,91]. Also, for human health, ABA plays an essential role in glucose metabolism, oxidative stress, tumor growth, ischemic retinopathies, and the central nervous system [92,93]. Furthermore, ABA showed an antimicrobial effect on *Helicobacter pylori* [94] and antiviral properties [95]. Bridi et al. [96] determined in *Brassica rapa* (*Brassicaceae*) and *Robinia pseudoacacia* BP (*Fabaceae*) 59.00 to 240.00 μg ABA/g BP. In another study [96], the ABA content varied from 25.60 to 355.50 μg ABA/g BP in multi-floral BP from plant species of *Asteraceae*, *Fabaceae*, and *Rosaceae* families.

In a recent study, Bayram et al. [44] reported the composition of their BBE samples, derived from *Asteraceae*, *Fabaceae*, *Plantaginaceae*, and *Rosaceae*, as gallic acid (0.34–3.47 μg/g BB), kaempferol (3.80–26.81 μg/g BB), quercetin (9.71–39.18 μg/g BB), and rutin (23.30–126.13 μg/g BB), values which were smaller than our results, except for rutin concentration. The results obtained in another study by Gercek et al. [80] on BP samples provided from *Asteraceae*, *Fabaceae*, *Campanulaceae*, *Cistacea*, and *Rosaceae* plant species also showed lower PC content values and similar rutin concentrations (115.42 μg/g BP) compared to the results obtained by us for the 12 samples of BB. The palynological profiles of the BB from this study varied, and it was challenging to differentiate the samples according to phenolic profiles and predominant families and correlate them with the preceding BB studies/research. However, the literature data confirm the PCs depicted in Table 2 [15,25,43,71,97,98]. The presence of PCs was also confirmed by FTIR analysis (Figure 4).

**Figure 5:**
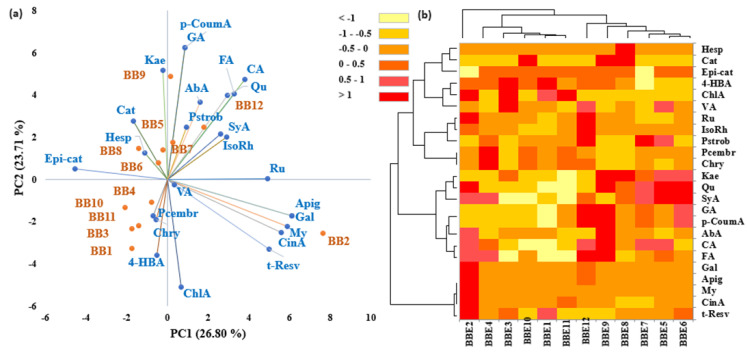
Discrimination of bee bread samples based on quantitative phenolic compound biomarkers: (**a**) principal component analysis and (**b**) hierarchical cluster and heat map analysis.

Multivariate statistical analysis, including principal component analysis (PCA) and heat map analysis (HMA), was applied to the phenolic quantitative data in order to differentiate between BB samples with different origins. From the PCA analysis, a clear discrimination of the BB2 sample was observed, which could be correlated with the botanical origin because this sample presented the highest percentage of the *Asteraceae* family plant species (Figure 2). Furthermore, apigenin (Apig), galangin (Gal), myricetin (My), cinnamic acid (CinA), t-resveratrol (t-Resv), and chlorogenic acid (ChlA) represent specific PCs for **BB2** and are distributed on the right-downside area of Figure 5a. Also, the right side of the figure indicates specific PCs for **BB7** and **BB12**, like rutin (Ru), isorhamnetin (IsoRh), syringic acid (SyA), pinostrombin (Pstrob), abscisic acid (AbA), ferulic acid (FA), caffeic acid (CA), quercetin (Qu), gallic acid (GA), and *p*-coumaric acid (*p*-CoumA). Corresponding to the palynological analysis, the **BB12** sample is specific to BP of plant species of *Amaranthaceae*, *Betulaceae*, *Bromeliaceae*, *Concolvulaceae*, *Lamiaceae*, *Onagraceae*, and *Violaceae*. The **BB7** sample also has plant pollen from the mentioned families, while presents a high content of pollen from the *Acanthaceae* family.

According to botanical origin (Figure 2 and Figure 3), **BB3**, **BB10,** and **BB11** present significant contents in pollen from plant species of *Amaryllidiceae*, *Cornaceae, Fabaceae*, *Fagaceae*, *Primulaceae*, *Resedaceae*, and *Salicaceae* families. Linking these results with Figure 5a, the pinocembrin (Pcembr), chrysin (Chry), and 4-hydroxybenzoic acid (4-HBA) are distributed to **BB1**, **BB3**, **BB4**, **BB10**, and **BB11**, which are clustered on the left downside of the PCA graph. Kaempferol (kae), catechin (cat), epicatechin (Epi-cat), and hesperidin (Hesp) represent specific PCs for **BB5**, **BB6**, **BB8,** and **BB9**, which are grouped on the left side of Figure 5a. Likewise, *Acanthaceae*, *Apiaceae*, *Asteraceae*, *Boranginaceae*, *Brassicaceae*, *Colchicaceae*, *Oleaceae*, *Onagraceae*, and *Pinaceae* were the families distinctive for these samples (as seen in Figure 3a). In particular, Hesp is linked to the *Ericaceae* plant species and is present only in **BB8** and **BB9**, which also have a significant content of phenolic acids and flavonoids.

The hierarchical clusters heat map confirms the PCA results, which highlight a discrimination of **BB2**. As seen in Figure 5b, the other BB samples are clustered into two main groups, corresponding to the left downside of the PCA graph and the upside.

The heat map of the PC profiles indicates a principal cluster, which corresponds to *t*-resv, CinA, My, Apig, and Gal, and the **BB2** sample, respectively. The main cluster is divided into two sub-clusters, which are distributed into other groups at the same distance.

A recent study [99] highlighted the considerable content in PCs of *Vaccinium* sp. (CinA, *p*-CoumA, CA, GA, SyA, Qu, etc.). Only the **BB8** sample had pollen from plants of the *Rutaceae* family, while Hesp was identified in its extract (**BBE8**). Hesperidin and its derivates are known to be found in the highest concentrations in citrus fruits (*Rutaceae* family) and in small amounts in *Mentha* sp. (*Lamiaceae*) [100]. A fact recently reconfirmed by Bakour et al. [101], who showed that BP with 50% *Citrus aurantium* had the highest hesperidin content among PCs (488.90 μg/g BP), and also showed more potent antioxidant activity.

### 3.3. Biological Activity of BBE

#### 3.3.1. Qualitative Assessment of the Antimicrobial Activity

**Figure 6:**
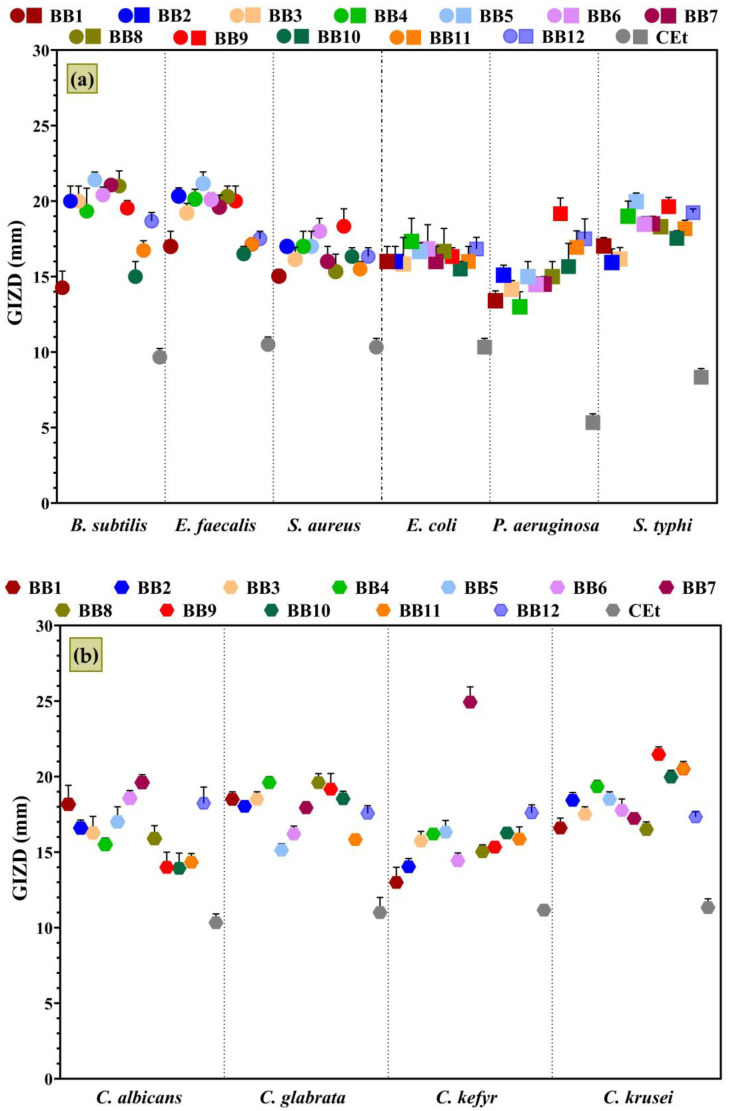
The growth inhibition zone diameters (GIZDs) of BBEs on selected pathogenic strains: (**a**) Gram-positive and Gram-negative bacteria; (**b**) yeasts. The significant influence of the BBEs on each microbial strain was statistically analyzed by one-way ANOVA and Tukey’s multiple comparisons tests. The *p*-value was <0.01 and the results were statistically significant.

Antimicrobial activity was qualitatively assessed by determining the diameters of the growth inhibition zones that appeared around the spot (of BBE samples) and expressing them as mean values ± SDs (Figure 6).

BBE samples presented a significant antimicrobial effect on the growth of all microbial strains tested, and *B. subtilis*, *E. faecalis*, *S. typhi*, *C. krusei*, and *C. glabrata* were the most sensitive strains.

The extract with the highest antibacterial activity on Gram-positive bacteria was **BBE5**, followed by **BBE8**, **BBE1**, **BBE2**, **BBE6**, and **BBE9**. The data obtained are linked to the botanical origins because these samples are also clustered in Figure 3. The susceptibility of Gram-positive bacteria to BBEs can be explained by the presence of flavonoids in high amounts, which, with their lipophilicity properties, damage the cell membrane (phospholipid bilayers) and inhibit the respiratory chain and ATP synthesis [102].

In the case of Gram-negative bacteria, **BBE5**, **BBE4**, **BBE9**, and **BBE12** showed the highest inhibitory effect. The antimicrobial profiles for yeasts differed, but **BBE7** and **BBE9** presented the most heightened sensitivity. Besides flavonoids, BBEs contained phenolic acids, which induced damage to the cytoplasmatic membranes of Gram-positive and Gram-negative bacteria, and microscopic fungi [103,104].

Quercetin, kaempferol, and caffeic acid were most likely the compounds responsible for the remarkable antimicrobial activity of the **BBE5** sample because these were present in the highest amount in this, have activity on both Gram-positive and Gram-negative bacterial strains, and the first two sometimes acted synergistically [105,106,107]. In addition, Qian et al. [108] revealed that vanillic acid, which had the highest concentration in this sample (**BBE5**), possessed antimicrobial activity against *S. aureus* and *E. coli*. These phenolic compounds were also present (in variable percentages) in other samples with significant antimicrobial activity (**BBE8**, **BBE1**, **BBE2**, **BBE6**, and **BBE9**).

Akhir et al. [109] reported that BB ethanol extracts presented more inhibitory effects against *B. subtilis* and *S. aureus* than *E. coli* and *S. typhi*. Also, Elsayed et al. [110] demonstrated the highest sensitivity for *S. aureus* (26 mm) and *B. subtilis* (24 mm) rather than *E. coli* (18 mm) and *C. albicans* (15 mm) due to the influence of citrus BB. The GIZDs determined by the *Rutaceae* plant BB are like the results in Figure 6 and can be correlated with the significant contents in quercetin, rutin, benzoic acid, and hesperidin [110,111]. The antifungal activity of BB has been little addressed until now, the diffusion assay being predominantly applied by Hudz et al. [112] and Elsayed et al. [110]. These two studies obtained growth inhibition diameters between 4 and 8 mm (*Candida albicans* CCM 8186, *Candida glabrata* CCM 8270, and *Candida tropicalis* CCM 8223) and 15 mm (*Candida albicans* ATCC 10221) for BB samples also extracted with ethanol. Still, according to our knowledge, there are no references to compare our results regarding the antimicrobial activity of BBE against *C. kefyr* and *C. krusei*.

#### 3.3.2. Quantitative Evaluation of Antimicrobial Activity

**Figure 7:**
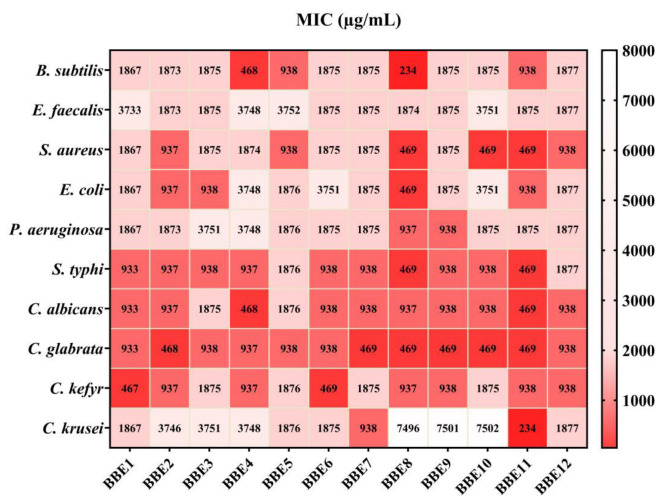
*Quantitative* evaluation of the antimicrobial activity of BBEs. Data results marked in intense red indicate the significantly lowest MIC values respectively a significant sensitivity of strains. The scale bar presents the variations in the sensitivity of tested strains from the highest (red) to the lowest (white).

The MIC value was characterized by the smallest concentration of the tested BBEs that inhibited microbial development. The results are shown in Figure 7, expressed as µg/mL.

Figure 7 highlights the most significant antimicrobial activity of BBEs on the pathogenic strains. *S. typhi* and *C. glabrata* were the most sensitive tested strains and, in contrast, *E. faecalis* was the most resistant to BBE.

The lowest MIC value was obtained for **BBE8** (234 µg/mL), followed by **BBE4** (468 µg/mL) on *B. subtilis*. Also, the extracts showed significant inhibition of *S. aureus* development, and **BBE8**, **BBE10**, **BBE11**, **BBE2**, and **BBE12** induced the highest sensitivity, a result somehow expected based on the known antimicrobial effects of caffeic acid, quercetin, and kaempferol against this strain. Hesperidin (together with other phenolic compounds) might have contributed to **BBE8**’s MIC value, given its higher antibacterial effect on Gram-positive than Gram-negative bacteria [113]. The multi-target antibacterial action mechanisms for *p*-coumaric acid suggested that it could also play an essential role in the fight against pathogenic bacteria, especially against *S. aureus* and *E. coli* [114].

In contrast, BBEs determined moderate sensitivity on *E. faecalis* and *P. aeruginosa*. *C. glabrata*, *C. albicans*, and *C. kefyr* were the least resistant to the action of the BBEs.

Overall, **BBE8**, **BBE9**, **BBE2**, and **BBE4** presented the highest antimicrobial activity, correlated with chemical composition (Figure 4, Table 1 and Table 2). Moreover, according to the statistical assays, the mentioned samples stood out from the others. In agreement with the PCA assays, **BBE2** was associated with precise taxonomic assignments (of plants with pollen contribution) and PCs. Also, **BBE12** is linked with FA, CA, and Qu, and with specific botanic families (Figure 3).

Abouda et al. [115] reported that BBEs inhibited *S. aureus*, *B. cereus*, *E. coli*, and *P. aeruginosa* at ½ and ¼ dilutions. Also, Urcan et al. [116] recorded the highest antimicrobial potential of Romanian BB for *S. aureus*. Another study [15] showed the significant antimicrobial activity of the BB originated from BPs of plants of the *Apiaceae* (35%), *Asteraceae* (24%), *Fagaceae* (16%), *Myrtaceae* (9%), *Punicaceae* (6%), and *Mimosaceae* (5%) botanical families. Bacterial strains *B. cereus*, *S. aureus*, and *L. monocytogenes* were sensitive to BBE, with 0.04–0.17 mg/mL and 0.08–0.35 mg/mL values for MIC and MBC (minimal bactericidal concentration), respectively. Comparable results were obtained for Gram-negative bacteria (*E. coli*, *E. cloacae*, and *S. typhimurium*), which were the least susceptible to BBE.

#### 3.3.3. Semiquantitative Assay of the Microbial Adherence to the Inert Substratum

**Figure 8:**
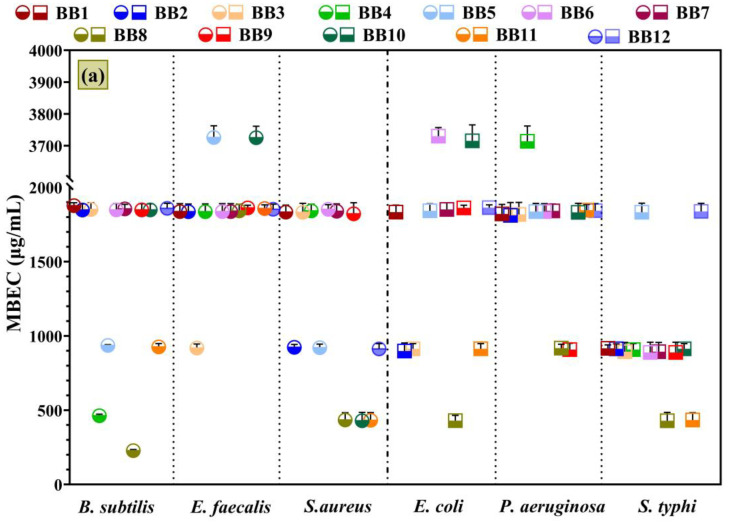

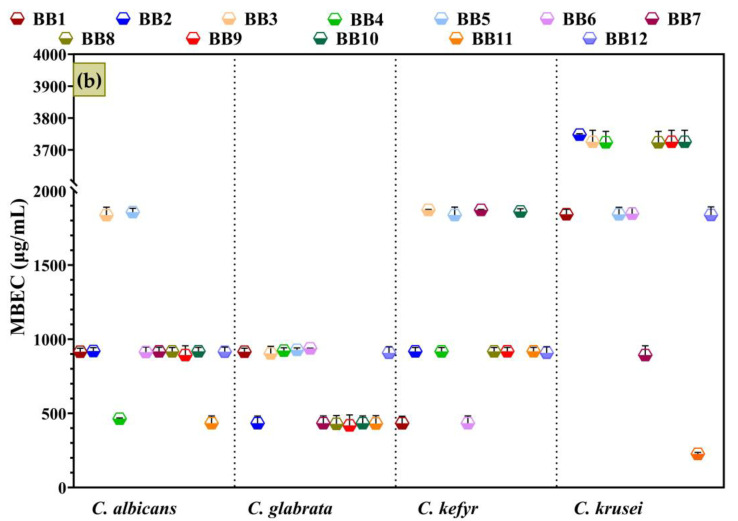
Graphical chart of the MBEC values: (**a**) Gram-positive and Gram-negative bacteria; (**b**) yeasts. The differences between BBEs were statistically analyzed using one-way ANOVA, followed by Tukey’s multiple comparisons tests.

The BBE’s influence on the tested microbial strains’ adherence to the inert substratum is displayed in Figure 8, which represents the minimal biofilm eradication concentration (MBEC) values.

Figure 8’s data confirm the qualitative (Figure 6) and MIC results (Figure 7) and are correlated with the chemical composition (Figure 4 and Table 2) and botanical origin of BBs (Figure 1 and Figure 2). Consequently, *S. typhi* and *C. glabrata* depicted the highest sensitivity in the presence of BBE samples. Also, BBEs significantly inhibited the adherence of *C. albicans* and *C. kefyr*. For the other strains, BBE samples showed similar antimicrobial profiles, but with some exceptions. For example, **BBE8**, with the highest TPC content, exhibited the strongest antibiofilm activity against *B. subtilis*. Also, a great inhibitory effect was displayed against *S. aureus*, *S. typhi*, *C. albicans*, and *C. kefyr*. Furthermore, **BBE2**, **BBE9**, and **BBE12**, which had a high amount of PCs, displayed significant antibiofilm effects on the tested strains. In addition, in any prior research, the BBs’ influences on adherence to the bacteria or fungi inert substratum were not investigated.

In agreement with the chromatographic results (Table 2), the representative flavonoids for BBEs are isorhamnetin, kaempferol, and quercetin, and their antimicrobial effects are reported in previous studies [117,118,119,120,121]. According to our knowledge, the antimicrobial properties of the isolated PCs fractions from BBEs were not studied due to limited data for BB. Additionally, the inhibitory effect of flavonoids (like quercetin and kaempferol glucosides) isolated from BP against *S. aureus*, *S. epidermidis*, *P. aeruginosa*, *E. coli*, *C. albicans*, *C. tropicalis*, and *C. glabrata* were reported [122].

**BBE8** and **BBE9**, with significant quercetin and kaempferol amounts, exhibited great antibiofilm effects against *P. aeruginosa* and *C. glabrata*. These samples had dominant pollen grains from the *Acanthaceae*, *Colchicaceae*, and *Ericaceae* botanical families. Moreover, in a recent study [28], *P. aeruginosa* was very sensitive to BB with a significant content of plant pollen from *Ericaceae* and *S. aureus* on BB from *Brassicaceae*; the results were correlated with the high TPC, TFC, and antioxidant activity of BP [123].

**BBE5** confirms these findings of antimicrobial activity on *B. subtilis*, *S. aureus*, and *C. glabrata* because it contains 31% *Brassicaceae* pollen. It is well known that the pollen from *Fabaceae* species is also associated with an antibacterial effect against *P. aeruginosa* [28].

In our study, the pollen from the *Fabaceae* family was present in all samples, but greater contents were found in **BBE10**, **BBE11**, and **BBE12**. In addition, **BBE10** had pollen predominantly from the *Salicaceae* family plant species, which demonstrated its antimicrobial potential [9]. Furthermore, **BBE2** had a significant abundance of pollen from *Asteraceae* family plants and the antimicrobial activity of *Achillea setacea*, *Antennaria dioica*, and *Helichrysum arenarium*, which are found in Romanian flora [124,125] was already proven. Also, **BBE2** discrimination is highlighted in the statistical assays of PCs (Figure 5).

Previous research [9] showed that BBEs exhibited greater antimicrobial activity than BPEs with pollen from similar plant species. Also, Pelka et al. [126] reported that BBEs presented a higher antimicrobial effect than BPEs on *S. aureus*, *S. epidermidis*, *E. coli*, and *P. aeruginosa*, with MIC values that ranged from 2.5 to 10% (*v*/*v*) and MBC from 2.5 to 5%. Additionally, Kaškanienè et al. [127] reported that the antibacterial, antifungal, and antioxidant activities increased after the fermentation of bee pollen with *Lactococcus lactis* and *Lactobacillus rhamnosus*. It is possible that certain metabolites of these strains may be responsible for antimicrobial activity in some cases. Despite incompletely understanding the inhibition mechanism of bacterial growth, resveratrol contribution can be significant, as well as other compounds that may be present in very low quantities under the detection limit of the applied method [33,128].

## 4. Conclusions

Bee bread is a promising source of phenolic compounds and antioxidants. The main objective of this study was to investigate the relationship between botanical origin, chemical composition, antioxidant activity, and the effect on selected pathogenic strains.

The palynological analysis revealed a high relative abundance of pollen from plants belonging to *Salicaceae*, *Asteraceae*, *Brassicaceae*, and *Acanthaceae* families. In total, thirty-one families were identified. In all bee bread samples, pollen “supplied” by plants from the *Fabaceae* family was always present, while the pollen of the least-represented species is part of the *Bromeliaceae*, *Convovulaceae* and *Rutaceae* families.

The flavonoid concentrations were much higher than the phenolic acids in all bee bread extracts analyzed. The extracts contained mainly rutin, hesperidin, and resveratrol, as well as a high content of quercetin, kaempferol, isorhamnetin, and abscisic acid. There were also significant quantities of *p*-coumaric acid, gallic acid, caffeic acid, and cinnamic acid. The **BBE2**, **BBE8**, **BBE9**, and **BBE12** samples had the highest levels of phenolic acids, flavonoids and heterosides. **BBE2** and **BBE9** presented the highest concentrations of phenolic compounds, which ranged from 9209.73 and 18,889.74 µg PC/g BB.

The antimicrobial activity of bee bread extracts is strongly linked to chemical composition, antioxidant activity, and pollen botanical origin. The bee bread extracts’ phenolic profiles are complex and different, and it is challenging to attribute the inhibitory growth effect to a single compound or a pollen type from a specific botanic family precisely. Furthermore, a synergistic effect between bioactive compounds is most probably responsible for the biological properties of bee bread.

The bee bread extracts presented a significant antimicrobial effect on the growth of all microbial strains tested. *Salmonella typhi* and *Candida glabrata* were the most susceptible tested strains. Also, *Candida albicans* and *Candida kefyr* were sensitive to the influence of bee bread extracts.

The present study reported the bee bread extracts’ antibiofilm effects/ inhibitory activity on microbial adhesion capacity to the inert substratum of bacteria or fungi for the first time. Likewise, the samples (**BBE8** and **BBE9**) that had pollen grains dominant from the *Acanthaceae*, *Colchicaceae*, and *Ericaceae* botanical families presented significant quercetin and kaempferol amounts and displayed great antimicrobial effects against *Pseudomonas aeruginosa* and *Candida glabrata*. In addition, the sensitivity of *Bacillus subtilis*, *Staphylococcus aureus*, and *Candida glabrata* is linked to pollen from *Brassicaceae* plant families (**BBE5**). Significant antimicrobial activity was correlated with pollen from plants belonging to the *Salicaceae* and *Asteraceae* families (**BBE10** and **BBE2**, respectively).

Rich in phenolic compounds and with significant antimicrobial properties, bee bread can be a valuable source of natural nutrients and bioactive compounds that enhance human health. Further studies should evaluate the pre- and probiotic potential of bee bread as well as the cytotoxic action to complement the existing data.

## Figures and Tables

**Table 1:** Total phenols content, total flavonoids content, and antioxidant activity.

Sample	TPC (GAE) ^1^	TFC (QE) ^2^	TEAC ^3^
**BBE1**	12.40 ± 0.010	**0.87 ± 0.502**	**0.07 ± 0.020**
**BBE2**	7.10 ± 0.005	0.52 ± 0.031	0.02 ± 0.010
**BBE3**	12.50 ± 0.005	**1.86 ± 0.516**	0.03 ± 0.005
**BBE4**	**14.40 ± 0.010**	0.53 ± 0.020	0.04 ± 0.006
**BBE5**	11.40 ± 0.005	0.50 ± 0.052	0.04 ± 0.009
**BBE6**	13.60 ± 0.005	0.60 ± 0.051	**0.06 ± 0.010**
**BBE7**	11.20 ± 0.025	0.45 ± 0.035	0.04 ± 0.004
**BBE8**	**18.30 ± 0.029**	0.52 ± 0.090	**0.05 ± 0.020**
**BBE9**	**15.80 ± 0.047**	0.59 ± 0.030	**0.05 ± 0.008**
**BBE10**	**18.30 ± 0.051**	0.70 ± 0.050	**0.05 ± 0.050**
**BBE11**	**14.90 ± 0.017**	**0.95 ± 0.011**	0.03 ± 0.030
**BBE12**	11.20 ± 0.015	**0.85 ± 0.500**	0.02 ± 0.005

^1^ TPC expressed as mg gallic acid equivalents/g BB; ^2^ TFC expressed as mg quercetin/g BB; ^3^ TEAC expressed as mmol Trolox/g BB. The red data represent the highest values. The differences between samples are statistically quantified using one-way ANOVA and Tukey’s multiple comparisons tests. The results are considered statistically significant (*p* < 0.0001).

**Table 2:** Phenolic compound content of BB (µg/g).

Phenolic Compound	Sample											
	BB1	BB2	BB3	BB4	BB5	BB6	BB7	BB8	BB9	BB10	BB11	BB12
**Phenolic acids**												
cinnamic acid	10.65 ± 0.13	**64.67 ± 0.30**	10.43 ± 0.14	8.65 ± 0.33	10.39 ± 0.21	15.98 ± 0.49	6.63 ± 0.28	7.65 ± 0.26	11.85 ± 0.34	9.91 ± 0.23	22.41 ± 0.12	12.58 ± 0.22
*p*-coumaric acid	58.02 ± 0.21	66.50 ± 0.52	67.10 ± 0.18	67.32 ± 0.78	73.29 ± 0.07	**84.19 ± 0.61**	79.09 ± 0.48	75.85 ± 0.51	**109.13 ± 0.45**	64.35 ± 0.12	69.43 ± 0.13	**98.71 ± 0.18**
ferulic acid	7.00 ± 0.14	27.20 ± 0.62	7.69 ± 0.27	29.27 ± 0.07	24.06 ± 0.40	14.85 ± 0.11	15.05 ± 0.40	14.38 ± 0.31	34.10 ± 0.36	16.94 ± 0.09	8.43 ± 0.06	**41.46 ± 0.11**
caffeic acid	52.27 ± 0.20	**76.76 ± 0.95**	54.41 ± 0.28	66.73 ± 0.98	**74.48 ± 0.27**	54.12 ± 0.62	**74.66 ± 0.27**	64.20 ± 0.43	**84.85 ± 0.38**	53.29 ± 0.43	55.28 ± 0.54	**76.90 ± 0.36**
chlorogenic acid	38.86 ± 0.54	40.60 ± 0.25	**54.10 ± 0.06**	N.D.	N.D.	N.D.	N.D.	N.D.	N.D.	N.D.	**53.94 ± 0.31**	N.D.
4-hidroxybenzoic acid	**50.22 ± 0.47**	47.73 ± 0.42	49.20 ± 0.11	47.32 ± 0.79	45.94 ± 0.74	46.29 ± 0.38	44.84 ± 0.88	46.73 ± 0.37	47.65 ± 0.24	47.59 ± 0.32	46.90 ± 0.35	47.37 ± 0.46
vanillic acid	30.84 ± 0.44	31.88 ± 0.16	39.39 ± 0.38	30.17 ± 0.40	34.73 ± 0.23	30.70 ± 0.40	30.64 ± 0.13	31.14 ± 0.69	30.66 ± 0.56	30.97 ± 0.14	29.27 ± 0.14	34.71 ± 0.27
syringic acid	30.32 ± 0.27	30.72 ± 0.35	30.19 ± 0.42	30.77 ± 0.76	30.84 ± 0.85	30.84 ± 0.17	30.73 ± 0.10	30.49 ± 0.38	30.30 ± 0.27	30.04 ± 0.06	30.07 ± 0.09	30.57 ± 0.09
gallic acid	58.02 ± 0.65	66.50 ± 0.40	67.10 ± 0.11	67.32 ± 0.66	73.29 ± 0.10	**84.19 ± 0.35**	79.09 ± 0.62	75.85 ± 0.30	**109.13 ± 0.56**	64.35 ± 0.26	69.43 ± 0.43	**98.71 ± 0.45**
**Flavonoids**												
catechin	N.D.	N.D.	N.D.	N.D.	N.D.	N.D.	N.D.	36.01 ± 0.58	34.54 ± 0.34	34.62 ± 0.17	N.D.	N.D.
epicatechin	51.48 ± 0.21	N.D.	51.25 ± 0.59	51.28 ± 0.35	51.23 ± 0.17	51.24 ± 0.16	N.D.	51.27 ± 0.20	51.28 ± 0.55	51.24 ± 0.24	51.33 ± 0.44	51.23 ± 0.56
myricetin	N.D.	45.07 ± 0.34	N.D.	N.D.	N.D.	N.D.	N.D.	N.D.	N.D.	N.D.	N.D.	N.D.
quercetin	**1001.36 ± 1.57**	**1178.45 ± 1.00**	**1012.16 ± 0.55**	**1014.32 ± 0.57**	**1182.06 ± 0.74**	**1188.93 ± 0.64**	**1160.44 ± 0.69**	**1121.08 ± 0.57**	**1192.04 ± 0.84**	**1007.52 ± 0.65**	**993.36 ± 0.32**	**1023.75 ± 1.02**
apigenin	30.88 ± 0.41	**322.38 ± 0.59**	42.20 ± 0.16	57.28 ± 0.46	36.88 ± 0.16	36.98 ± 0.52	35.85 ± 0.42	44.48 ± 0.34	47.45 ± 0.54	49.86 ± 0.28	32.15 ± 0.22	88.94 ± 0.24
galangin	7.58 ± 0.14	**341.12 ± 0.45**	20.66 ± 0.89	37.88 ± 0.62	14.58 ± 0.42	14.69 ± 0.30	13.38 ± 0.04	23.24 ± 0.34	26.66 ± 0.23	29.41 ± 0.14	9.16 ± 0.32	74.14 ± 0.45
kaempferol	**816.86 ± 1.37**	**984.68 ± 1.17**	**1266.22 ± 0.64**	**831.16 ± 0.55**	**1449.53 ± 0.44**	**1513.51 ± 0.57**	**1354.52 ± 0.65**	**1655.62 ± 0.92**	**1892.27 ± 1.17**	**810.46 ± 0.67**	**762.24 ± 0.76**	**943.44 ± 0.33**
isorhamnetin	**466.10 ± 1.44**	**671.82 ± 1.40**	**530.69 ± 0.37**	**587.13 ± 0.69**	**523.09 ± 0.37**	**523.95 ± 0.95**	**532.70 ± 0.74**	**543.26 ± 0.76**	**540.78 ± 0.87**	**464.43 ± 0.34**	**485.41 ± 0.59**	**1333.09 ± 0.60**
chrysin	65.95 ± 0.45	65.59 ± 0.23	63.09 ± 0.44	82.79 ± 0.27	63.57 ± 0.21	66.43 ± 0.17	64.35 ± 0.42	62.65 ± 0.24	62.28 ± 0.32	69.76 ± 0.26	68.83 ± 23	69.54 ± 0.54
pinocembrin	52.65 ± 0.61	52.66 ± 0.45	50.18 ± 0.20	67.87 ± 0.54	52.39 ± 0.33	53.60 ± 0.10	52.28 ± 0.45	50.96 ± 0.74	50.68 ± 0.26	54.55 ± 0.34	55.19 ± 0.40	52.57 ± 0.21
pinostrombin	64.70 ± 0.40	64.60 ± 0.57	64.40 ± 0.38	65.23 ± 0.58	65.78 ± 0.30	64.27 ± 0.14	67.17 ± 0.57	64.43 ± 0.38	64.50 ± 0.37	64.83 ± 0.78	65.01 ± 0.72	67.70 ± 0.75
**Heteroside Flavonoids**												
rutin	66.76 ± 0.89	**332.03 ± 0.35**	84.19 ± 0.20	**113.74** ± 0.30	61.22 ± 0.16	91.78 ± 0.20	56.51 ± 0.52	72.50 ± 0.55	76.89 ± 0.45	99.65 ± 0.65	76.27 ± 43	**383.49 ± 0.57**
hesperidin	N.D.	N.D.	N.D.	N.D.	N.D.	N.D.	N.D.	**52.17 ± 0.21**	N.D.	N.D.	N.D.	N.D.
**∑ phenolic acids, flavonoids, and heterosides**	**2960.54**	**4510.98**	**3564.65**	**3256.25**	**3867.34**	**3966.54**	**3697.90**	**4123.97**	**4497.02**	**3053.76**	**2984.11**	**4528.91**
**Stilbenoide**												
resveratrol	77.54 ± 0.61	79.51 ± 0.51	77.29 ± 0.42	76.77 ± 0.39	76.82 ± 0.35	77.41 ± 0.28	76.80 ± 0.78	76.86 ± 0.34	76.41 ± 0.45	76.45 ± 0.14	76.54 ± 0.54	76.54 ± 0.13
**Sesquiterpene**												
abscisic acid	783.39 ± 0.54	**4619.24 ± 0.85**	**2344.76 ± 0.75**	379.04 ± 0.48	352.79 ± 0.85	718.90 ± 0.42	**2088.84 ± 1.26**	**2241.88 ± 1.33**	**14,316.31 ± 0.78**	547.64 ± 0.62	190.51 ± 0.38	276.63 ± 0.40
**Total**	**3821.47**	**9209.73**	**5986.70**	**3712.07**	**4296.95**	**4762.84**	**5863.54**	**6442.72**	**18,889.74**	**3677.84**	**3251.17**	**4882.08**

N.D. (not detected). Data marked in bold and red/green represent each identified compound’s highest/lowest values. The data colored in black and bold represents the significant content in the BB’s PC. The background of cells colored in red represents the largest amounts of PC.

## Data Availability

Data are contained within the article.
